# Quinine

**Published:** 1897-06

**Authors:** 


### Quinine.

It is reported that certain manufacturers are making an attempt to have a duty put on quinine. At the present time it is about 50 cents per ounce. Before the duty was removed it sold for $4 to $5 per ounce. We hope that every medical journal in the country will instruct the Representatives for their District to retain it on the free list. Quinine has become almost an absolute necessity to the American people. It would be rank injustice to again place a duty upon it.—*Pacific Medical Journal.*

